# The concept analysis of helplessness in nurses during the COVID‐19 pandemic: A hybrid model

**DOI:** 10.1002/nop2.1955

**Published:** 2023-07-20

**Authors:** Tooba Hoseini Azizi, Jamalodin Begjani, Alireza Arman, Akram Sadat Sadat Hoseini

**Affiliations:** ^1^ School of Nursing and Midwifery Tehran University of Medical sciences Tehran Iran; ^2^ School of Nursing and Midwifery Tehran University of Medical Sciences Tehran Iran; ^3^ School of Nursing and Midwifery Tehran University of Medical Sciences: The member of research centre of Quran, Hadith and Medicine of Tehran University of Medical Sciences Tehran Iran

**Keywords:** concept analysis, COVID‐19, helplessness, hybrid model, nurses

## Abstract

**Aim:**

‘Helplessness’ is one of the psychological concepts that exploring nurses' helplessness during the COVID‐19 pandemic can lead to timely intervention and empowerment of nurses.

**Design:**

Concept development.

**Methods:**

It was carried out using Schwartz‐Barcott and Kim's hybrid model.

**Results:**

In the literature review, helplessness is characterized by anxiety symptoms (muscle tension, headache, anorexia and insomnia) and, to some extent, depression (loneliness, guilt, apathy and insensitivity). In the fieldwork, five categories were obtained, including antecedents of helplessness (the nature of the disease, professional responsibility, personal lifestyle disruption and social behaviours), attributes of helplessness (inability to do more for the patient, inability to control the situation, feeling of uselessness, frustration, giving up and uncertainty to continue), consequences of helplessness (increase clinical error, physical manifestations and psychological manifestations), strategies to reduce feelings of helplessness and the difference among helplessness, powerlessness and hopelessness.

**Conclusion:**

Nurses' helplessness occurs when the nurse has no control over the situation and cannot change it.

## 1 INTRODUCTION

‘Helplessness’ is one of the psychological concepts that nurses experience, especially in the face of patients suffering from serious illnesses (Back et al., 2015). The concept of helplessness in nursing care is breaking down, following the loss of control over the world around’ (Back et al., 2015). Researchers have conducted numerous studies on different populations of patients and their families, nurses and physicians to better understand the sense of helplessness (Back et al., 2015; Clements & Cummings, 1991; Farrell, 1989; Mehta & Ezer, 2003; Meier et al., 2001; Milberg et al., 2004; Olofsson et al., 2003; Samuelsson et al., 2002; Tarzian, 2000). The results show that ‘helplessness’ has different somatic, emotional and cognitive manifestations depending on the individual's perspective and experiences (Back et al., 2015). In caring for dying patients, nurses experience ‘helplessness’ due to a lack of control over the situation (Tarzian, 2000) or a feeling of inadequacy for the patient (Farrell, 1989). The feeling of inability to help further relieve the physical stress of dying children and the psychological stress of their families are perceived by nurses as a feeling of ‘helplessness’ (Olofsson et al., 2003). Physicians feel helpless in response to increasing patient dependence and the physician's need for more time, especially when the patient requests that the physician cannot fulfil (Meier et al., 2001).

In psychology, ‘helplessness’ is confirmed by a person's verbal report, indicating discouragement, dissatisfaction, pessimism about the future, crying, boredom and guilt (Renan Alves et al., 2017). In the past, the concept of ‘helplessness’ in psychology has been defined by two theories 'learned helplessness' and 'helplessness‐hopelessness’. In the theory of learned helplessness, Seligman et al. said ‘helplessness’ is experienced when a person realizes that his or her response has no effect on the outcome, and as a result of this cognitive error, the motivation to respond is weakened. In the theory of ‘helplessness‐ hopelessness’, anxiety and depression with the feeling of ‘helplessness’ are described as expecting negative consequences in the future that are uncontrollable (Seligman, 1975).

Nurses' experiences of helplessness differ from explaining this concept in the form of theories (Drew, 1990), and to date, a clear definition and its characteristics have not been provided in nursing. In addition, there is a conceptual overlap of helplessness in scientific texts with other psychological concepts such as powerlessness and hopelessness (Pan & Chiou, 2004). While each concept of powerlessness and hopelessness is related to a particular state of mind and describes different sensations, ‘helplessness’ occurs when a person believes that necessary action has been taken, resulting in a feeling of inability to work harder and maintain energy. According to the latest NANDA nursing diagnoses, definitions and classification, hopelessness is defined as ‘the feeling that one will not experience positive emotions or an improvement in one's condition’, and powerlessness is ‘a state of actual or perceived loss of control or influence over factors or events that affect one's wellbeing, personal life, or the society’. (Herdman et al., 2021).

During the COVID‐19 pandemic, healthcare providers' workloads have increased. In the meantime, nurses ignoring their needs have accepted their moral and professional responsibility in caring for patients. Wearing personal protective equipment during long working hours can lead to physical stress (Ornell et al., 2020; Sun et al., 2020; Tan et al., 2020; White, 2021). In addition, the feeling of insecurity against infection and transmission to family members, observing the suffering of patients in the absence of the family and the family's suffering away from the patient's bedside have caused severe psychological stress for nurses (Allahverdipour, 2020; Hacimusalar et al., 2020). Studies have shown that when nurses have close contact with patients with emerging infectious diseases, they experience loneliness, anxiety, fear, fatigue, sleep disorders and other physical and mental health problems (Nelson & Lee‐Winn, 2020; Sun et al., 2020).

In caring for COVID‐19 patients, feelings of helplessness have been described as the inability to help patients and families, thinking about continuing the disease indefinitely and frustration with wearing personal protective equipment (Allahverdipour, 2020). All in all, the nurses faced a situation that was painfully unknown to them. Most of them had enthusiastically and selflessly started caring for the patients, hoping that their efforts would pay off and they would soon return to society in good health, but they faced a situation that was indescribable and exhausting for them and in their evaluations. They did not meet the desired results and had not reached according to the provision of their services. The health services providers did not have a precise definition of this phenomenon and did not know its dimensions and cause scientifically and clearly, and they needed to know this new situation well. The community providing health services needed to receive sufficient support and be empowered in this situation, and this was not possible without identifying all aspects of the helplessness phenomenon created in the care of patients with COVID‐19. Helplessness can threaten the nurse's physical and mental health and affect the quality of care and his/her physical and mental health. The new experience of nurses' helplessness in dealing with the COVID‐19 pandemic needs a more in‐depth study and a more appropriate definition. Accurate and timely diagnosis of helplessness as an abstract psychological response to acute conditions in nurses during the COVID‐19 pandemic can lead to timely intervention and empowerment of nurses who demonstrate the characteristics of this concept. Therefore, we conducted this study to define helplessness and clarify it in nurses during the COVID‐19 pandemic.

## 2 PRACTICAL GOALS

By clarifying the nurses' understanding of helplessness, a clear and precise definition of this concept can be provided in the nursing of COVID‐19 patients. This definition makes it easier to understand the situations that nurses refer to as helplessness, avoids using other psychological concepts and introduces appropriate design tools to measure it. On the other hand, the need to prevent this phenomenon becomes apparent in the healthcare team, and the way to design prevention and support programmes for nurses becomes clear.

## 3 METHODS

This study aims to develop a concept of helplessness in nurses during the COVID‐19 pandemic using Schwartz‐Barcott and Kim's hybrid model.

This concept has some definitions in nursing and psychology literature, but in COVID‐19, it was necessary to define the new dimensions revealed about this concept in the real environment created due to the disease. In such situations, one of the appropriate methods is the hybrid conceptualization to provide an introduction for deeper and more studies. This model is a way to create, develop and expand concepts, especially in nursing, and consists of three phases, theory (literature review), fieldwork and the final analysis (Schwartz‐Barcott, 2000).

## 4 THEORETICAL PHASE

**FIGURE 1:**
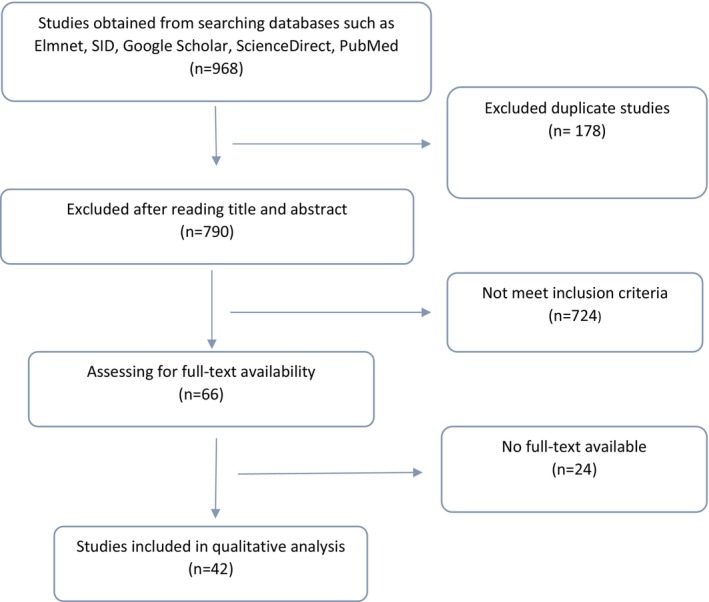
The overall flowchart of the literature review.

We conducted a literature review focusing on the conceptual definition and measurements of helplessness in nursing. The overall flow chart of the literature review is shown in Figure 1.

To explain the concept of helplessness in nursing based on existing literature, we first searched for helplessness's meaning in dictionaries to explore the concept's fundamental meaning. Second, we searched databases such as PubMed, EMBASE, CINHAL, Web of Science and PsycINFO using the keywords helplessness and nurse COVID‐19. The literature search was conducted in October 2021. Studies with the keywords used in titles and abstracts, published in English and available in full text without publication year restrictions were included. We managed founded literature through the EndNote program. The literature review focused on the definition and measurement of the concept in nursing personnel or population related to nursing professions like physicians, patients and their families. We found 968 studies. One hundred seventy‐eight of them were excluded due to duplication. Of the 790 studies, 724 were excluded for not meeting the inclusion criteria. The full‐text availability of the remaining 66 studies was checked; for 24 studies, we could not access full texts, leaving the final 42 studies selected for the literature review.

## 5 FIELDWORK PHASE

The fieldwork phase aims to confirm and purify a concept by developing and integrating the analysis started in phase 1 with ongoing empirical observations done in this phase. This phase includes four steps: selecting a research environment, entering into negotiations, selecting participants and collecting and analysing information (Schwartz‐Barcott, 2000).

We selected the participants with a purposeful sampling method from a referral hospital for COVID‐19. Bachelor's degree‐level nurses with at least 3 of work experience in the COVID‐19 pandemic who are willing to participate in the study and able to provide informed consent entered to the study.

Due to following physical distance rules during the pandemic, we conducted in‐depth interviews using video calls (WhatsApp) and phone calls. Observational information on the clinical settings, which could not be collected through phone calls, was gathered through questions. Ten nurses participated based on their experience providing care to COVID‐19 patients and observing the maximum variation in gender, age and work experience. All nurses had a history of being infected with COVID‐19 (Table 2).

Data were collected using in‐depth semi‐structured individual interviews. At the beginning of this phase, an initial interview guide was prepared, which helped the researcher to ask more questions to explore the area. The interviews began with an open‐ended question: ‘Tell us about your first care experience with COVID‐19’. Other questions were then asked based on the interview guide and the participants' answers, such as ‘How do you feel when taking care of COVID‐19 patients? Has your feeling changed during the care of these patients so far? What name do you give to your feelings when caring for these patients? Tell me about the worst moment you experienced while caring for these patients’. Also, exploratory questions were used to continue the interview, such as ‘Can you explain more? Alternatively, “When you say …, what do you mean?”’. At the end of each interview, participants were asked to state if they had any other concerns. The interviews were conducted between December 2021 and January 2022; each was 45 and 60 minutes long. The characteristics of age, sex, ward, work experience duration and history of nurses and their families got COVID‐19 were gathered using a demographic questionnaire. Data collection and analysis were performed simultaneously by the researchers. Data analysis was done using the qualitative content analysis method of Lundman & Graneheim (22). In this way, transcribed interviews were read several times to get a general understanding, and then semantic units were extracted from them, which later were coded. Similar codes were classified in the same classes or subcategories. The subcategories were arranged according to their semantic proximity, and a suitable title was given to similar subcategories, forming the main categories. Data analysis did use MAXQDA software Ver 10. Data collection occurs until saturation has been achieved when no new themes have emerged from the participants (Speziale et al., 2011).

Lincoln and Goba's criteria (credibility, transferability, dependability and confirmability) ensured data quality (Lincoln & Guba, 1985). In this regard, the researchers tried to increase the credibility of the findings by establishing appropriate interaction and in‐depth interviews with the participants, immersion in data, interviewing the maximum variety of participants, writing field notes, peer checking the analyses and coding. A review by participants was also used. All research documents were carefully presented and recorded, including operational notes (basic information from the recording, field notes and memos) and theoretical notes (guidance questions, interview form and informed consent) to increase conformability.

## 6 FINAL ANALYTIC PHASE

The theoretical phase findings, experimental observations and qualitative data are integrated. Their results were compared, analysed and integrated (Bousso et al., 2009). In this phase, we paid attention to the practical situation of helplessness to make a useful definition that fits COVID‐19. All categories in the previous phases were compared and analysed in relevance to yield themes that seemed supported through literature and fieldwork data (Ide‐Okochi & Tadaka, 2016). The analytical approach of this phase helps to redefine the concept. We scanned the definition dragged from the theoretical phase approved in the fieldwork phase, what characteristics were in the theoretical phase but could not be verified in the fieldwork phase and what characteristics were not in the theoretical phase but verified in the fieldwork phase. Through this process, we found similarities and differences between the two phases and analysed them; then, we defined the concept with emphasis on the COVID‐19 situation.

## 7 ETHICAL CONSIDERATIONS

The Research Ethics Committee has approved this study of the Research Council of Tehran University of Medical Sciences with the ethics code IR.TUMS.MEDICINE.REC.1399.334. Participants were given informed oral consent to participate in the study and recording interviews and were assured that their information would remain confidential.

## 8 FINDINGS

### 8.1 Theoretical phase

#### 8.1.1 Helplessness definitions

The meanings of helplessness and helpless in the Merriam‐Webster dictionary are lacking protection or support, marked by an inability to act or react and being unable to be controlled or restrained (Merriam‐Webster). In medical terminology, helplessness is defined as a ‘state of incapacity, vulnerability, or powerlessness associated with the perception that one cannot do much to improve a negative situation that has arisen’ (Merriam‐Webster, 2021).

#### 8.1.2 Helplessness and related concepts in nursing

The oldest definition of helplessness in nursing is related to ‘hopelessness and helplessness’, published in 1964 (Shea & Hurley, 1964). Two nurses named Shea and Harley defined hopelessness as ‘the feeling that any attempt to positively change a patient's condition is doomed to failure before it is made’ and helplessness as ‘The belief that everything that can be done has been done, which leads to an inability to conserve energy and make an effort’. (Shea & Hurley, 1964).

Carlson and Blackwell defined helplessness in 1970 as ‘a complex syndrome of emotions, thoughts, and behaviors that occurs when events are out of control’. In other words, everything that can be done has been done, and no action significantly affects the result. Helplessness is the feeling of drowning due to losing control over the surrounding world (Carlson & Blackwell, 1978). In 1985, Clifford defined the concept of helplessness in nursing care as ‘the feeling of being broken by losing control of the surrounding environment’ (Clifford, 1985).

In 1990, Drew explored the concepts of helplessness, hopelessness and powerlessness based on Erickson's theory and explained their differences. He proposed hope versus hopelessness, purpose and skill versus powerlessness and willpower and self‐control versus helplessness (Drew, 1990; Pan & Chiou, 2004). Studies showed that helplessness would have different somatic, emotional and cognitive manifestations depending on the individual's perspective and previous experiences, but participants in all studies have experienced helplessness as feeling useless due to a lack of control over the situation (Back et al., 2015; Meier et al., 2001; Tarzian, 2000).

Helplessness is an inevitable experience for nurses and physicians who work with severe illnesses (Back et al., 2015). There are two models of ‘less involvement’ in physicians and ‘excessive involvement’ in nurses in the helplessness experience. While the physician withdraws and behaves passively and insensitively, nurses feel pressured, anxious and even frustrated because the professional structure puts them in a position of ‘unauthorized responsibility’, which means nurses feel responsible for alleviating the suffering caused by the patient's medical decision while not being allowed to change or challenge the decision (Back et al., 2015).

The findings of the review of studies of helplessness in other populations related to nursing, including patients and their families, are summarized in Table 1.

**TABLE 1:** Characteristics of the concept of helplessness in reviewed studies published before COVID‐19.

Author and year	Study population	Concept properties
Clements & Cummings, 1991	Nurses caring for patients with pain	The inability to provide comfort to clients with pain caused nurses to feel helpless with frustration characteristics, leading to the avoidance of patients and apathy
Farrell, 1989	Nurses and families of dying patients	Not being helpful to the loved one in the final time led to a sense of helplessness, resulting in guilt in nurses and families
Olofsson et al., 2003	Paediatric nurses	The lack of time for optimal care of patients makes nurses experience emotions of frustration, powerlessness, hopelessness and inadequacy because they feel unable to influence the situation
Tarzian, 2000	Patients with respiratory distress	Nurses responded to the patient's loss of control and their own sense of helplessness by avoiding patients suffering from air hunger
Meier et al., 2001	Physicians of seriously ill patients	Physicians developed a sense of helplessness related to patients increasing dependency and demands on the physician's time, and against the impossible request and began to avoid patients
Milberg et al., 2004	Relatives of patients in palliative care	Helplessness concerned the next of kin's perception of the patient's suffering and the next of kin's own feelings of insufficiency and resulted in both physical and psychological symptoms, such as muscle tension, headache, loss of appetite, anxiety and depression. Helplessness is related to feelings of guilt, anger and loneliness.
Samuelsson et al., 2002	Fathers experienced stillbirth	Fathers felt inadequate to protect their partners and experienced helplessness.
Farrell, 2002	Families of patients with ovarian cancer	Family members experienced helplessness due to not being helpful to the loved one in the final time.
Lindholm et al., 2002	Significant others of patients with breast cancer	Significant others' experienced helplessness when they could not influence the situation during the patient's suffering.
Mehta & Ezer, 2003	Spouses of patients with pain	Spouses described helplessness when witnessing their loved one in pain, which is tied to a sense of loss of control, uselessness and the inability to influence the situation

**TABLE 2:** Demographic and occupational information of the participants.

Variables	Participant information
Age (mean ± standard deviation)	30.4 ± 6.71 years
Work experience (mean ± standard deviation)	5.74 ± 3.21 years
Position	Clinical nurses: 7
	Head nurses: 3
Wards	Infectious disease wards: 3
	intensive care unit: 2
	Coronary care unit: 2
	Emergency: 3

**TABLE 3:** Characteristics derived from the concept of helplessness in the theoretical phase and fieldwork phase.

	Theoretical phase	Fieldwork	Analytical phase
Antecedences	Inability to provide comfort to clients with pain (Clements & Cummings, 1991) Not to be helpful to the loved one in the final time (Farrell, 1989) Perception of patient's suffering (Gordon et al., 2021; Lindholm et al., 2002; Mehta & Ezer, 2003; Milberg et al., 2004; Tarzian, 2000) Patients’ increasing dependency and demands on the physician's time, and against the impossible requests (Meier et al., 2001) Fears of disease recurrence (Ferrell et al., 2002) COVID‐19 knowledge and intervention deficit, the need to wear personal protective equipment (PPE) and fear of disease transmission to family and friends (Gordon et al., 2021; Ornell et al., 2020; Sun et al., 2020; Tan et al., 2020)	The nature of the disease	The problems and challenges in nursing care related to nature of disease
	Lack of time for ‘meticulous care’ for patients (Olofsson et al., 2003; Sun et al., 2020; White, 2021) Heavy workload (Kellogg et al., 2021; Ornell et al., 2020; Sun et al., 2020; Tan et al., 2020)	Professional Responsibility	Insufficient professional responsibility
	When the nurses were unable to meet their physical and mental needs (Gordon et al., 2021; Ornell et al., 2020; Sun et al., 2020; Tan et al., 2020) Social isolation (Gordon et al., 2021; Ornell et al., 2020; Sun et al., 2020; Tan et al., 2020)	Personal lifestyle disruption	Social and family isolation Lifestyle disruption
		Social behaviours	
Attributes	Inability to act or react. Not able to be controlled or restrained and incapacity (American Psychological Association, 2021; Merriam‐Webster) Unable to influence the situation (American Psychological Association, 2021; Lindholm et al., 2002; Mehta & Ezer, 2003; Olofsson et al., 2003)	Inability to do more for the patient	Unable to be effective for patient Incapacity
	Feelings of loss of control (Ferrell et al., 2002; Kellogg et al., 2021; Mehta & Ezer, 2003; Tarzian, 2000)	Inability to control the situation	Loss of control
	Feeling uselessness (Mehta & Ezer, 2003; Tan et al., 2020) Feeling of insufficiency, inadequacy or ineffectiveness (Gordon et al., 2021; Milberg et al., 2004; Samuelsson et al., 2002; Tan et al., 2020; White, 2021)	Feeling uselessness	Feeling uselessness
	Frustration (Clements & Cummings, 1991; Shaw, 2020; Tan et al., 2020)	Feeling frustration Giving Up	Frustration
	Thinking about leaving the field (Shaw, 2020)	Uncertainty to continue	Indetermination
Consequences	Avoidance of patients (Clements & Cummings, 1991; Meier et al., 2001; Tarzian, 2000) Absence of response (Olofsson et al., 2003) Affect the quality of care (Tan et al., 2020)	Threaten safety	Safety threats
	Stress, irritability, physical and mental fatigue and despair (Ornell et al., 2020) Muscle tension, headache, loss of appetite, sleeplessness, anxiety/worry, depression, feeling of loneliness and anger (Milberg et al., 2004; Sun et al., 2020) Anxiety, dependence, withdrawal and demoralization (American Psychological Association, 2021) Apathy (Clements & Cummings, 1991) Guilt (Farrell, 1989; Milberg et al., 2004) Self‐blame (Tan et al., 2020) Crying (Shaw, 2020)	Physical manifestations (cold hands and feet, weakness, dizziness, inability to stand, heart palpitations, sweating, loss of appetite, weight loss and disturbed sleep) Psychological manifestations (anxiety, boredom, sadness, crying, restlessness, grumbling, bad manners, anger, apathy, homesickness, talkativeness, laughing less, not enjoying activities and feeling oppressed)	Physical and psychological changes

#### 8.1.3 Helplessness scales

The search for a tool to measure *helplessness* in different populations and nurses had no results. Existing tools measure the phenomenon of *learned helplessness* in the patients or caregivers. (Lovibond & Lovibond, 1995; Omachi et al., 2010; Quinless & Nelson, 1988).

#### 8.1.4 Nurses' helplessness in the COVID‐19 pandemic

The significant increase in the hospitalization of patients during the COVID‐19 pandemic had a significant impact on nurses. Nurses experienced anxiety, depression, post‐traumatic stress disorder and unresolved grief (White, 2021). Nurses caring for patients with COVID‐19 frequently reported feeling a sense of *helplessness* (Kellogg et al., 2021). Qualitative studies of nurses' experiences during the COVID‐19 pandemic revealed that nurses were more likely to report feelings of helplessness; when nurses felt that their efforts for the patients were not enough (while they wanted to help the patients more, it was not possible), and when the nurses were unable to meet their physical and mental needs. In addition, increased workload, lack of information about the disease, the need to wear personal protective equipment (PPE), fear of disease transmission to family and friends and social isolation have caused loneliness and *helplessness* in nurses (Gordon et al., 2021; Ornell et al., 2020).

In a phenomenological study, Sun et al. showed that nurses report fear of making mistakes or not doing enough for patients as a precondition for negative emotions such as anxiety and *helplessness*; failing to meet their physical and psychological needs brought a sense of *helplessness* (Sun et al., 2020). In the study on front‐line nurses' experiences treating COVID‐19 patients, nurses stated that the feeling of uselessness in nursing care has contributed to *helplessness* and *powerlessness* (Tan et al., 2020). In another study, nurses experienced a deep sense of helplessness when they felt that their learning and experiences in the nursing profession could not help the patients. They inevitably observed the patient's suffering and could not do anything (Gordon et al., 2021). Nurses expressed wanting to do more to help their patients and described how it was often beyond their control to change their conditions (Kellogg et al., 2021). In White's study, nurses described having to give up the ‘meticulous care’ they usually provided and only being able to do the ‘basics’ for patients causing them to feel helpless (White, 2021). Medical students at the forefront of COVID‐19 treatment have expressed frustration and helplessness with phrases such as ‘I want to cry’ and ‘I think about leaving the field every day’. The researcher states in this article that if they ignore the problem of helplessness and ways to reduce it in these students, they will suffer from ‘learned helplessness’ in the next phase and emphasizes that efforts should be made to prevent it as much as possible (Shaw, 2020). Virtual critical care^1^ nurses have played a vital role in assisting patient care in intensive care units in the United States for many years. In the COVID‐19 pandemic, the need for telenursing has also highlighted the role of this type of nursing care. Arneson et al. point to another aspect of these nurses' sense of *helplessness*; those VCC nurses experience fear and *helplessness* when guiding clinicians through a virtual camera. Nurses could only support the patient's emotional needs through the camera when the patient was in urgent need of care and reassure the patient that help would be forthcoming, but they felt helpless because they could not do more (Arneson et al., 2020).

### 8.2 Fieldwork phase

The findings of the fieldwork phase were classified into five main categories: antecedents of helplessness, attributes of helplessness, consequences of helplessness, strategies to reduce feelings of helplessness and the difference among helplessness, powerlessness and hopelessness. Characteristics derived from the concept of helplessness in the theoretical phase and fieldwork phase are shown in Table 3.

Antecedents of helplessness:

The antecedents of helplessness were categorized into four subcategories: the nature of the disease, professional responsibility, personal lifestyle disruption and social behaviours. A female nurse defined the strange and unknown nature of the disease in this way:

We did not know what would happen to the patient in 2 min, and this was very strange … the distance between life and death was very short … while the patient was fully awake, talking, and drinking fluids, he/she was not in bed tomorrow … and it bothered me mentally, especially if he/she was young.

Another nurse mentioned her sense of professional responsibility in the COVID‐19 pandemic and said:

We had no choice but to adapt to the situation … because there was no more workforce and co‐workers who wanted to support us and cover our shifts so that we could have a short rest … There was no other way.

The COVID‐19 pandemic has disrupted the lifestyle of nurses, as one of the participants described the situations that made her feel helpless:

Helpless means that my husband and I are sick, and I have a 4‐year‐old child at home, for whom I had to prepare different food and put it in a separate room. My son wanted us to hug him … I had to go on shifts, too.

The increase in the number of patients due to non‐observance of health protocols made nurses helpless:

We were helpless. We were tired because we saw that some people did not follow the health protocols, the number of patients was increasing day by day, our wards were all full, and our colleagues were getting infected one by one.

#### 8.2.1 Attributes of helplessness

The fieldwork results showed that nurses' attributes of helplessness during the COVID‐19 epidemic included the inability to do more for the patient, inability to control the situation, feeling of uselessness, frustration, giving up and uncertainty to continue. One of the critical care nurses experienced helplessness in this situation:

In the face of this disease, when I could not help the patient, I felt helpless … One was dying in front of my eyes, and I could not do anything.

The patient's non‐response to nursing care made the nurse feel helpless and inadequate:

The patient who had come was breathing, talking, and what happened was that suddenly his condition got worse in an hour or two! … We felt uselessness, we felt empty.

We saw that the patient was getting worse day by day. You do whatever you must do, but it does not affect the patient's recovery, causing a feeling useless.

Some participants described helplessness as a feeling of frustration and giving up and said:

My co‐workers were frustrated … I was sitting and crying. We really could not do anything … The patients were alert; we could see their respiratory distress until the last moment, and we could not do anything.

I am a nurse, and my wife is also a nurse; for fear of transmitting to others, my wife was isolated, and I was alone with my son for four months; I did not leave my son alone anywhere … I tried to keep my spirits up and take care of my life and my child, but sometimes I gave up.

The participants also experienced the feeling of helplessness in deciding whether to continue nursing, leave the job or regret choosing the nursing profession:

Now I prefer not to go to work. I think about it a lot. I think about it once or twice a day. However, I say if I do not go, they say that he/she was afraid because of Corona's condition, gave up, and did not come. There will be a bad history or a bad memory, or I will later regret why I left my job.

#### 8.2.2 Consequences of helplessness

Helplessness leads to physical and psychological manifestations and increases medical errors.

Nurses expressed the physical and psychological symptoms they experienced when they felt helpless:

My feelings are involved; I had tachycardia, my hands and feet were cold, and I had a state like this.

My colleagues were being harassed and getting physical symptoms … For example, you saw one of them taking sick leave this week and another next week. It was for busy shifts, working conditions, patients' conditions, clothes, family, and COVID‐19 stress.

I was very nervous and tired. I would get upset very soon; my colleagues stated that your morals have changed for a while, and you will get angry soon! While I had a gentle personality and tried to treat everyone well.

Nurses caring for COVID‐19 patients believe that a sense of helplessness threatens the safety of nurses and patients. Not wearing personal equipment due to helplessness threatens the nurse's safety, and reducing care accuracy endangers the patient's safety:

When you are helpless, it affects eighty to 90 percent of your work. You are registering the medicine incorrectly… It even affects patient care and reduces motivation to work.

We felt helpless…, we said let it be whatever it wanted; we really could not stand these clothes anymore. It is not easy to work in these conditions.

#### 8.2.3 Strategies to reduce feelings of helplessness

Nurses tried to manage their feelings of helplessness with the help of hope, self‐encouragement, music and nature, cooperation in providing care and giving meaning to life:

If the feeling of helplessness prevails, it will cause less life expectancy and more harm. I tried to keep my spirits up with hope for the future.

I tried to be alone with myself for a few minutes, sitting in a secluded corner and talking to myself, comforting myself in the hope that my unlike feeling would end, and returning to work when I was somewhat hopeful and encouraged.

When my work shift was over and I was coming home, I was bored. I wanted to be alone, listen to music, be calm, or I would like to go to nature to vent my discomfort.

Now we work more interactively; for example, last night, when I was helpless and could not work, my colleagues registered my patients' laboratory tests, HIS^2^ And instead, I Collected all blood samples for laboratory testing in the morning.

I will not let that feeling of helplessness overwhelmed me. I have my methods. I do not consider these conditions to be the end of the matter because I believe that whatever happens, there must be some charity in you.

#### 8.2.4 The difference among helplessness, powerlessness and hopelessness

According to participant's experiences, helplessness differs from powerlessness and hopelessness. In this category, the distinctions of these concepts were included. A participant explained the difference between experiencing powerlessness and helplessness as follows:

When my body gets tired of doing a heavy activity, I experience powerlessness that goes away with one to two hours of rest, but when I feel helpless, I am mentally and emotionally tired, and it may bother me for 2‐3 weeks to get out of my mind.

Participants experienced differences between helplessness and hopelessness:

When you feel helpless, when you see that everything you do is not working for her, hope may be very dim, but you still have hope.

### 8.3 Findings from the final analysis

According to the literature review, helplessness is a mental concept and a negative psychological feeling that nurses experience when they cannot comfort patients while perceiving their suffering. During the COVID‐19 pandemic, disease knowledge, intervention deficit, wearing personal protective equipment (PPE), fear of disease transmission and lack of time for ‘meticulous care’ for patients during heavy workload lead to nurses experiencing helplessness in the care of COVID patients. Additionally, nurses' likelihood of feeling helpless has increased because they could not meet their physical and mental needs and lived in social isolation for a long time.

In helplessness, the person feels unable to help themselves or anyone else. When the nurses feel unable to influence the situation and have no control over it, care is not helpful to the patient and feeling of uselessness, insufficiency, inadequacy, ineffectiveness and frustration, they feel helpless and think about leaving the field repeatedly.

Helplessness causes numerous physical and psychological symptoms. A helpless nurse experienced anxiety symptoms (muscle tension, headache, loss of appetite and sleeplessness) and, to some extent, depression (loneliness, guilt, crying, self‐blame, apathy and irritability). These physical and psychological changes cause avoidance of patients, absence of response and affect the quality of care. Helplessness differs from powerlessness and hopelessness, which in some cases have overlapped with these concepts.

In the second phase of the study, the results of the fieldwork showed that the antecedents of helplessness emergence during the COVID‐19 pandemic include the nature of the disease, professional responsibility, personal lifestyle disruption and social behaviours. These findings are along with the results of the first phase in aspects of patient care, professional responsibility and personal lifestyle disruption affecting the occurrence of helplessness in nurses. The social behaviours that lead to nurses' helplessness were not found in the literature.

Attributes of nurses' helplessness in the COVID‐19 epidemic include the inability to do more for the patient, inability to control the situation, feeling of uselessness, frustration, giving up and uncertainty to continue. These findings confirm the results of the first phase.

There was no difference between the consequences of the helplessness found in the first and second phases. A helpless nurse experiences anxiety and depression symptoms. During the COVID‐19 pandemic, nurses who experienced helplessness stated that their and their patients' safety had been threatened.

Most nurses try to control feelings of helplessness by maintaining hope, supporting colleagues, caring for themselves and taking refuge in spiritual beliefs. Some nurses also try to eliminate this feeling by taking extended sick leave or requesting resignation. In the first phase, no results were found to compare with the findings of the second phase of this study, which shows the strategies of nurses to manage helplessness during the COVID‐19 pandemic. In phase 2 of this study, nurses repeatedly used powerlessness and helplessness and compared the two concepts. According to their experience, helplessness is much more enduring than powerlessness, does not decrease with rest and there is no relationship between workload and the intensity of feeling helpless. They believed that powerlessness was more tolerable than helplessness. Also, nurses referred to maintaining hope while being helpless. In helplessness, nurses are discouraged from improving the condition, but they are not hopeless. With hope, they believe that they will feel better in the future. Thus, they actively sought new information about how to care for and treat COVID‐19 disease. The differences and overlapping of these three concepts were also found in the first phase of this study.

## 9 DISCUSSION

Regarding the antecedents of helplessness, nurses felt helpless by observing non‐compliance with health protocols that increased the number of patients. According to this finding, Brazilian and Italian nurses, tired and helpless from caring for the large number of patients with COVID‐19, have appealed to all citizens through social media, asking them to ‘stay home’ and follow public health recommendations (Fontanini et al., 2021; Forte & Pires, 2020). Forcing nurses to adhere to professional responsibilities has been reported as another cause of helplessness. Nurses had to return to work despite not fully recovering from their illnesses because they needed to cover shifts as their colleagues became infected and the number of patients increased. In previous health crises, nurses have experienced feelings of isolation and helplessness in the face of the stress of high‐intensity work (O'Boyle et al., 2006). The subcategory of personal lifestyle disruption deals with the fact that nurses, in addition to enduring high physical and psychological stress at work, also did not have the opportunity to be comfortable at home. Concerned about transmitting the infection, they changed their clothes, bathed frequently and even came home with masks. As each family member became infected with COVID‐19, nurses saw themselves as the cause of transmission and felt remorse and conscience stricken. Nurses severely limited their social interaction with their family and friends and endured psychological pressures in isolation. In a review of the psychological experiences of caregivers of patients with COVID‐19, Sun et al. reported that failure to meet nurses' physical and psychological needs caused them to feel helpless. All nurses expressed concern about the risk to their family's health, stressing that family members were also concerned about their health. Nurses who chose to be isolated felt helpless and guilty after being separated from their families. Nurses also reported fatigue, discomfort, helplessness from high‐intensity work and difficulty protecting themselves. Due to the unknown nature of the disease, patients' care and response to treatment are different. Nurses have reported concerns about medical malpractice or not taking adequate care of patients as a source of negative emotions such as anxiety and helplessness (Sun et al., 2020). In the COVID‐19 pandemic, in addition to increased workload, lack of information on how to transmit, manage and treat the disease, the need to wear personal protective equipment (PPE), fear of transmitting the disease to family and friends and subsequent social isolation of nurse's caused loneliness and helplessness in nurses (O'Boyle et al., 2006; Ornell et al., 2020; Sun et al., 2020) – observing the sufferings of patients and families who had been far apart have also caused severe psychological stress, including a sense of helplessness for nurses (Nelson & Lee‐Winn, 2020; Sun et al., 2020). An antecedent of helplessness that was presented in the articles but did not appear in the results of the fieldwork phase is that nurses feel trapped in the decisions of physicians, patients and families and feel responsible for alleviating the suffering caused by the patient's medical decision, while not being allowed to change or challenge the decision (Back et al., 2015).

To the findings of the second category of attributes of helplessness, nurses' uncertainty to continue nursing or to resign in situations where they and their families were at risk was identified. Feelings of doubt and hesitation have been identified as an essential component of anxiety (Back et al., 2015) and as one of the underlying factors of feelings of helplessness (Sand et al., 2008). Regarding the subcategory of feelings of powerlessness, Tarzian (2000) showed that nurses of dying patients experienced ‘helplessness’ due to the impossibility of administering more narcotics at the patient's request to reduce pain and attributed it to the inability to control the condition (Tarzian, 2000). Nurses of dying children perceive helplessness as the inability to help further relieve children's physical stress (Olofsson et al., 2003). Helplessness has also been experienced due to the inability to provide patient care in telenursing care models (Arneson et al., 2020). About the subcategory of feeling uselessness, nurses have reported ‘helplessness’ in caring for dying patients in the form of feelings of inadequacy. Also, based on the experiences of front‐line nurses in treating COVID‐19 patients, the feeling of uselessness in patient care has been the antecedent of a sense of inability and frustration (Tan et al., 2020).

The feelings of fraction and diminution expressed by the nurses' speeches are standard terms in the Persian language, equivalent to helplessness and inability. The English equivalent of these two terms is to give up. Seligman often refers to the term give up as an example of helplessness (Seligman, 1975). In introducing the concept of helplessness in physicians and nurses, Becket al. have used the word give up as a cognitive manifestation of helplessness (Back et al., 2015). Because fraction and diminution signify surrender and despair, the researcher asked them to describe the feeling in detail when the participants used the terms. Data analysis revealed that participants meant the experience of helplessness, not frustration. Feelings of uselessness and inability are attributes of helplessness experienced by nurses and patients. The feeling of uncertainty in the patient's experience of helplessness differs from the nurse's and is more due to the ambiguity of the disease prognosis. Nurses have doubts about accepting care for patients with COVID‐19 and continuing or resigning from their jobs. However, in both cases, this hesitation and uncertainty lead to feelings of helplessness and anxiety.

Most of the symptoms of anxiety and, to some extent, depression can be diagnosed by assessing the physical and psychological manifestations of nurses' helplessness. Alves Silva et al. have shown that during helplessness, depressed moods such as symptomatic discouragement and dissatisfaction, and pessimism about the future, crying, boredom, guilt and self‐despair appear (Renan Alves et al., 2017). In a study by Shaw et al., medical students at the forefront of COVID‐19 treatment also expressed feelings of hopelessness and helplessness with phrases such as ‘I want to cry’ (Shaw, 2020).

Findings of theoretical and fieldwork in confirmation of each other showed that while expressing the experience of helplessness, nurses did not accept the hopelessness of changing their condition or that of patients. In a study by Taylor et al., one of the front‐line nurses stated this in the treatment of COVID‐19: ‘I feel helpless and depressed right now. I know I'm brave, so I always remind myself I will get better’. In our study, nurses supported each other in caring and self‐care methods to cope with feelings of helplessness, mainly sitting alone in the natural environment and music. Recourse to spiritual beliefs has been reported as a way to relieve nurses of helplessness. Most nurses stated that the first conduct to alleviate their helplessness was to leave work and be alone. As the results of Back et al. (2015) study show, the first reaction of nurses and physicians to helplessness is to remain silent and try to avoid it (Back et al., 2015).

About distinguishing helplessness from the powerlessness and hopelessness category, other studies pointed out that these concepts overlap, as in the study of Milberg et al. (2004) and Clements and Cummings (1991) that the concepts of powerlessness and helplessness were used synonymously (Clements & Cummings, 1991; Milberg et al., 2004). In 1990, Drew examined the concepts of helplessness, hopelessness and powerlessness separately based on Erikson's theory and explained their differences. Considering their conceptual proximity, he proposed their opposite concepts: hope versus despair, purpose and skill versus disability and willpower and self‐control versus helplessness (Drew, 1990). Some features of helplessness extracted from the results of the fieldwork reflect the definition of powerlessness in NANDA nursing diagnoses, definitions and classification. NANDA defines powerlessness as ‘a state of actual or perceived loss of control or influences over factors or events …’ (Herdman et al., 2021). In this study, uncertainty in role‐play, depression, insufficient participation in care, lack of control and guilt are common features of helplessness and powerlessness. There is also a fine line between the characteristics of helplessness and hopelessness. In NANDA's nursing diagnoses, definitions and classification, the attributes of hopelessness include a change in sleep patterns, loss of appetite, expression of words indicating discouragement and insufficient involvement in care (Herdman et al., 2021) which are consistent with the attributes of helplessness found in this study.

Generally, nurses experience helplessness during the COVID‐19 pandemic, similar to before, especially in attributes and consequences. Most of the differences are in the antecedents of helplessness. In non‐pandemic conditions, the nature of the disease (e.g. being chronic and incurable) had the most significant impact on the incidence of nurses' helplessness (Clements & Cummings, 1991; Farrell, 1989; Olofsson et al., 2003). While during pandemics, the significant effects of the disease on nurses' personal lives, their confrontation with professional and moral conflicts and even people's social behaviours were important antecedents of helplessness in nurses. The results of the fieldwork show that the consequences of nurses' helplessness correspond to the characteristics of anxious and somewhat depressed moods. Nurses used self‐control and willpower techniques as positive defence mechanisms against feelings of helplessness and expressed hope during this phenomenon.

## 10 CONCLUSION

*Nurses' helplessness* is a mental concept and is a negative psychological feeling that has the characteristics of anxiety and depression. It occurs when the nurse feels he/she has no control over the situation and cannot change it, her knowledge and care are not helpful for the patients and she/he cannot help the patient further. Helplessness is a feeling that many nurses experience during the COVID‐19 pandemic. Challenges in nursing care related to the nature of the disease and heavy workload caused a sense of insufficient professional responsibility in nurses. Also, nurses experienced social and family isolation and lifestyle disruption during COVID‐19. With such conditions, they feel helpless in the forms of feeling unable to be effective for the patient, incapacity, loss of control, uselessness, frustration and indetermination. Changes occur in the nurse's physical and psychological state, along with the feeling of helplessness, which threatens the safety of herself/himself and her/his patients.

Therefore, diagnosing the clinical manifestations of *helplessness* makes it possible to treat the diagnosed cases, manage human resources and reduce harm to staff. Moreover, on the other hand, with timely intervention, its adverse effects on patients can be prevented. Given the importance of the issue and the fact that this type of *helplessness* may occur in other medical groups that interact directly with the patient, similar research is recommended.

## 11 LIMITATIONS

This study was conducted during the COVID‐19 pandemic, which limited face‐to‐face interviews due to health protocols. The interviews were conducted by telephone or online (WhatsApp), so collecting observational data from clinical settings was impossible.

## FUNDING INFORMATION

This study was not funded by any institute and was supported by the Tehran University of Medical Sciences (TUMS) with grant no: 99‐2‐101‐49387.

## CONFLICT OF INTEREST STATEMENT

None.

## Data Availability

Data sharing is not applicable to this article as no datasets were generated or analysed during the current study.

## Endnotes

^1^
Virtual critical care (VCC)

^2^
‐ Health Information System (HIS)
